# JAK inhibitors and adverse cardiovascular events: Class effect or molecule-specific risk?

**DOI:** 10.3389/fphar.2026.1896980

**Published:** 2026-09-08

**Authors:** Mislav Radić, Tina Bečić, Petra Šimac Prižmić, Marija Rogoznica Pavlović, Marijana Vučković, Josipa Radić, Josip Anđelo Borovac, Almir Fajkić, Andrej Belančić

**Affiliations:** 1 Department of Internal Medicine, Division of Rheumatology, Allergology and Clinical Immunology, University Hospital of Split, Split, Croatia; 2 Internal Medicine Department, University of Split School of Medicine, Split, Croatia; 3 Cardiovascular Disease Department, University Hospital of Split, Split, Croatia; 4 Hospital for Medical Rehabilitation of Heart and Lung Diseases and Rheumatism “Thalassotherapia-Opatija”, Opatija, Croatia; 5 Department of Internal Medicine, Division of Nephrology and Dialysis, University Hospital of Split, Split, Croatia; 6 Department of Pathophysiology, Faculty of Medicine, University of Sarajevo, Sarajevo, Bosnia and Herzegovina; 7 Department of Basic and Clinical Pharmacology with Toxicology, Faculty of Medicine, University of Rijeka, Rijeka, Croatia

**Keywords:** baricitinib, cardiovascular, filgotinib, Janus kinase inhibitors, major adverse cardiovascular events, tofacitinib, upadacitinib

## Abstract

Janus kinase inhibitors (JAKis) have established themselves as a valuable treatment option in treating rheumatoid arthritis and other immune-mediated inflammatory diseases, providing rapid and effective disease control across multiple refractory populations. However, their cardiovascular safety has come under intense scrutiny following the ORAL Surveillance trial, which showed an increased risk of major adverse cardiovascular events (MACE; a composite of cardiovascular death, non-fatal myocardial infarction, and non-fatal stroke) with tofacitinib compared with tumor necrosis factor inhibitors in patients with elevated baseline cardiovascular risk. Randomized trials, observational studies, and pharmacovigilance analyses involving tofacitinib, baricitinib, upadacitinib, and filgotinib have produced heterogeneous findings, with no consistent replication of a uniform class-wide cardiovascular signal. This divergence has generated ongoing debate about whether the observed risk reflects a true JAK inhibitor class effect or is driven by molecule-specific properties, patient selection, and study design. Mechanistic plausibility exists for both hypotheses through shared JAK–STAT pathway effects on vascular inflammation and thrombosis. This narrative review synthesizes current evidence from clinical trials, real-world data, and regulatory perspectives to critically assess the available evidence regarding whether cardiovascular risk represents a class effect or a molecule-specific phenomenon.

## 1 Introduction

### 1.1 Clinical context and therapeutic importance

Rheumatoid arthritis (RA) is a chronic systemic autoimmune disease (Scott et al., 2010) with a substantial and increasing global burden. Its incidence and prevalence are projected to rise in the coming decades, particularly among women (Cai et al., 2023). Despite these trends, RA-associated mortality has declined, reflecting earlier diagnosis and the widespread adoption of treat-to-target strategies using conventional synthetic, biologic, and targeted synthetic disease-modifying antirheumatic drugs (Black et al., 2023; Hansildaar et al., 2021). Nonetheless, cardiovascular disease remains the leading cause of death in RA and a major contributor to excess mortality (Hansildaar et al., 2021).

The therapeutic landscape has expanded with the introduction of Janus kinase inhibitors (JAKis), oral agents that modulate intracellular cytokine signaling through the Janus kinase-signal transducer and activator of transcription (JAK–STAT) pathway (Hu et al., 2021). Tofacitinib (TOFA), baricitinib, upadacitinib (UPA), and filgotinib differ in their selectivity for JAK isoforms, a feature with potential implications for both efficacy and safety (Antonioli et al., 2024). As their use has broadened in clinical practice, attention has increasingly shifted from efficacy to safety, particularly cardiovascular outcomes (Konzett V., 2025).

### 1.2 Why cardiovascular risk matters

Given this substantial cardiovascular burden, cardiovascular risk reduction has become an integral component of comprehensive RA management (England et al., 2018). Major adverse cardiovascular events (MACE), comprising cardiovascular death, myocardial infarction, and stroke, serve as the primary clinical endpoints for assessing cardiovascular outcomes in this population (Ytterberg et al., 2022). Patients with RA have a substantially elevated baseline risk for MACE compared with the general population, driven by chronic systemic inflammation, endothelial dysfunction, and traditional risk factors such as dyslipidemia, hypertension, and smoking (England et al., 2018; Weber et al., 2023). Anti-inflammatory therapy, including biologic and targeted synthetic disease-modifying agents, can mitigate this risk by suppressing systemic inflammation and slowing atherogenesis. Paradoxically, certain immunomodulatory treatments may inadvertently increase cardiovascular risk through off-target effects, such as alterations in lipid profiles, coagulation pathways, or blood pressure (Ytterberg et al., 2022; Atzeni et al., 2021). This duality underscores a therapeutic paradox: while effective disease control is expected to improve cardiovascular outcomes, specific mechanisms of immune modulation may simultaneously predispose patients to adverse events. Understanding this balance is critical when evaluating the safety profile of novel therapies such as JAKis, particularly in high-risk RA populations, and highlights the need for vigilant cardiovascular monitoring and individualized risk assessment in clinical practice (Ytterberg et al., 2022).

### 1.3 The safety signal that changed the field

The cardiovascular safety of JAKis came under increased scrutiny after the Oral Rheumatoid Arthritis (ORAL) Surveillance trial, a randomized, open-label, post-marketing noninferiority study comparing two doses of TOFA (5 mg and 10 mg twice daily) with tumor necrosis factor inhibitors (TNFi) (adalimumab (ADA) or etanercept) in patients with RA aged 50 years or older and at least one additional cardiovascular risk factor. Over a median follow-up of 4 years, TOFA did not meet noninferiority criteria for MACE and malignancies. The incidence of MACE was higher with TOFA than with TNFi, identifying a clinically relevant safety signal in a high-risk population (Ytterberg et al., 2022).

These findings led to major regulatory actions, including boxed warnings from the US Food and Drug Administration (FDA) (FDA, 2021) and restrictions from the European Medicines Agency (EMA), extending caution to the JAKi class despite limited molecule-specific evidence (EMA, 2022). As a result, prescribing patterns have become more restrictive, especially in patients with elevated cardiovascular risk.

**FIGURE 1:**
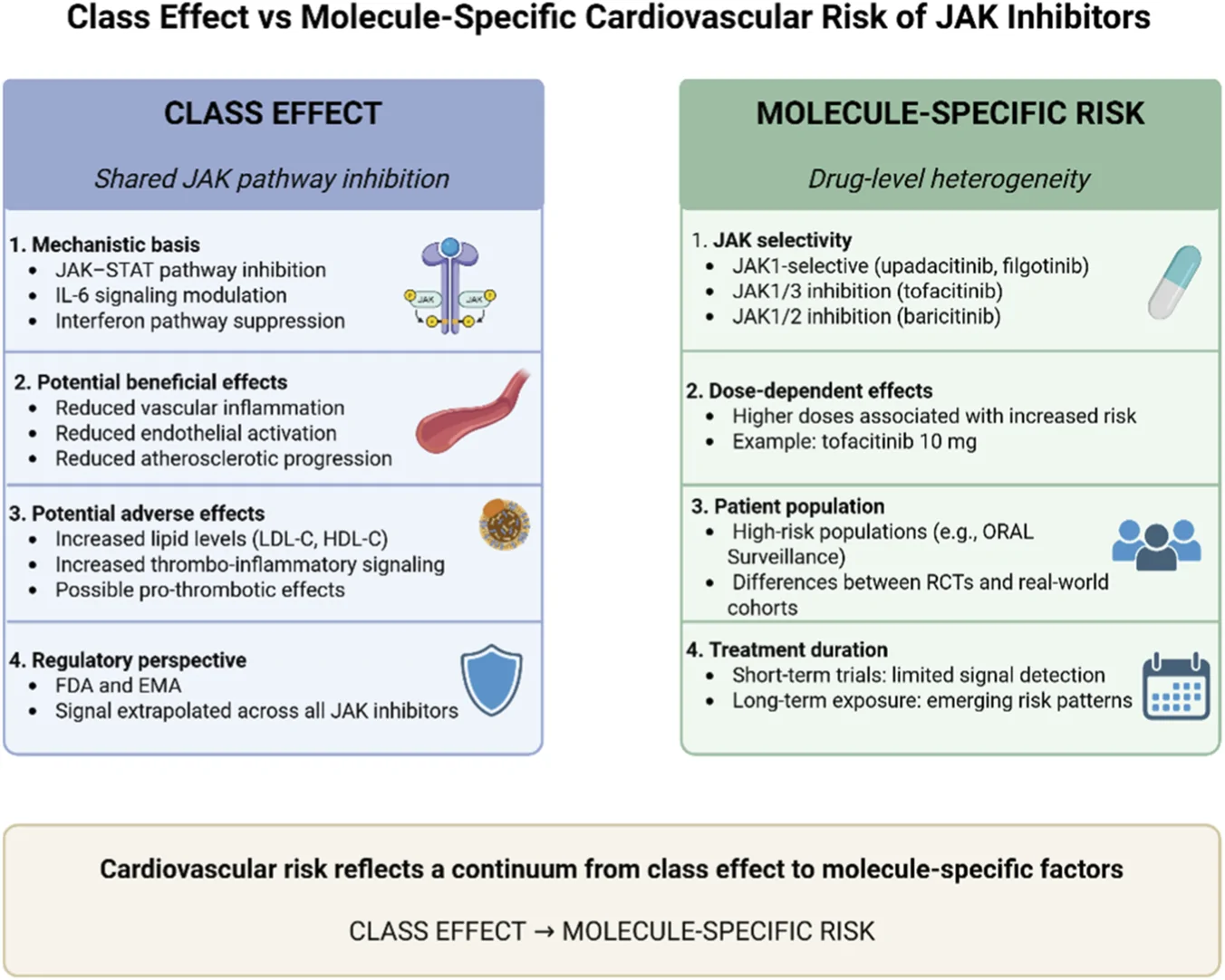
Class Effect vs. Molecule-Specific Cardiovascular Risk of JAK Inhibitors. Created in BioRender. Vučković, M (2026) https://BioRender.com/ezd2qd0. Abbreviations: JAK, Janus kinase; STAT, signal transducer and activator of transcription; IL-6, interleukin-6; LDL-C, low-density lipoprotein cholesterol; HDL-C, high-density lipoprotein cholesterol; FDA, U.S. Food and Drug Administration; EMA, European Medicines Agency; RCTs, randomized controlled trials; CV, cardiovascular.

Whether this signal reflects a class effect of JAK inhibition or a molecule-specific risk remains an open and clinically important question (Figure 1). This narrative review aims to critically appraise mechanistic evidence, randomized trial data, and real-world studies to clarify the cardiovascular safety profile of individual JAKis and inform risk-stratified therapeutic decision-making.

## 2 Methods

This article was designed as a narrative review to integrate current mechanistic, clinical, translational, real-world, regulatory, and guideline-based evidence regarding the cardiovascular safety of JAKis in patients with RA. Given the complexity of cardiovascular risk assessment in RA and the rapidly evolving evidence surrounding JAK inhibition, the primary objective was to critically synthesize available data into a clinically relevant framework addressing the ongoing question of whether cardiovascular risk represents a class effect of JAK inhibition or varies among individual agents. A narrative review was considered the most appropriate methodological approach because this topic encompasses heterogeneous evidence derived from molecular and translational studies, randomized controlled trials, long-term extension studies, observational cohorts, pharmacovigilance analyses, meta-analyses, and regulatory recommendations, making formal quantitative synthesis inappropriate.

A targeted literature search was conducted using PubMed and Scopus. The search included publications from 1 January 2017 through 5 April 2026, corresponding to the period during which evidence from pivotal phase III trials, long-term extension studies, observational cohorts, pharmacovigilance analyses, and regulatory evaluations substantially expanded the understanding of the cardiovascular safety of JAKis in RA. To maximize retrieval of relevant publications, manual screening of reference lists from systematic reviews, meta-analyses, narrative reviews, clinical practice guidelines, regulatory documents, consensus statements, and original research articles was also performed.

Earlier landmark publications published before 2017 were included selectively when considered essential for establishing the biological rationale and historical context of the review, including foundational studies on the JAK–STAT signaling pathway, vascular biology, inflammation-driven atherosclerosis, endothelial dysfunction, thrombosis, and cardiovascular risk associated with RA.

Searches combined Medical Subject Headings (MeSH), controlled vocabulary, and free-text terms related to rheumatoid arthritis, JAK inhibition, and cardiovascular outcomes. Representative search terms included: “rheumatoid arthritis,” “Janus kinase inhibitor,” “JAK inhibitor,” “JAK-STAT,” “tofacitinib,” “baricitinib,” “upadacitinib,” “filgotinib,” “cardiovascular disease,” “cardiovascular safety,” “cardiovascular risk,” “major adverse cardiovascular events,” “MACE,” “myocardial infarction,” “stroke,” “cardiovascular mortality,” “venous thromboembolism,” “pulmonary embolism,” “deep vein thrombosis,” “atherosclerosis,” “endothelial dysfunction,” “vascular inflammation,” “lipid metabolism,” “thrombosis,” “platelet activation,” “real-world evidence,” “observational study,” “registry,” “pharmacovigilance,” “post-marketing surveillance,” “clinical trial,” “FDA,” “European Medicines Agency,” “EULAR,” and “ACR.”

Titles and abstracts were initially screened for relevance, followed by full-text evaluation of potentially eligible publications. Articles were considered eligible if they fulfilled one or more of the following criteria: (i) investigated biological mechanisms linking JAK inhibition with cardiovascular physiology or pathology, including vascular inflammation, endothelial function, lipid metabolism, thrombosis, hematopoiesis, or atherosclerosis; (ii) evaluated cardiovascular outcomes, major adverse cardiovascular events, venous thromboembolism, or other cardiovascular safety endpoints during treatment with JAK inhibitors in patients with RA; (iii) reported evidence from randomized controlled trials, long-term extension studies, observational cohort studies, registry analyses, comparative effectiveness studies, pharmacovigilance databases, or post-marketing surveillance; (iv) synthesized evidence through systematic reviews or meta-analyses; or (v) provided regulatory recommendations, clinical practice guidelines, or expert consensus relevant to cardiovascular risk assessment and therapeutic decision-making in patients receiving JAK inhibitors.

Greater emphasis was placed on randomized controlled trials, large observational studies, systematic reviews, meta-analyses, and international regulatory documents because these provide the highest level of evidence regarding clinical cardiovascular safety. Mechanistic investigations were primarily used to support biological plausibility, explain potential differences among individual JAKis, and facilitate interpretation of clinical findings. Publications not available in English and studies unrelated to RA, JAKis, or cardiovascular outcomes were excluded.

The relevance and inclusion of publications were determined through consensus among the authors based on their complementary expertise in rheumatology, cardiovascular medicine, internal medicine, epidemiology, clinical pharmacology, and pathophysiology. Discrepancies regarding study eligibility or interpretation were resolved through discussion until consensus was achieved.

The narrative synthesis was organized into five interconnected domains: (i) biological mechanisms potentially linking JAK inhibition with cardiovascular risk; (ii) cardiovascular safety evidence from randomized clinical trials of individual JAKis; (iii) real-world evidence, observational studies, registries, and pharmacovigilance data; (iv) regulatory actions and contemporary guideline recommendations; and (v) integration of available evidence addressing the ongoing debate regarding class effect versus molecule-specific cardiovascular risk and its implications for individualized therapeutic decision-making.

Particular emphasis was placed on integrating mechanistic evidence with clinical trial findings and real-world data to provide a balanced interpretation of current knowledge. Areas of uncertainty—including differences among JAKi selectivity profiles, variability in study populations, limited statistical power for rare cardiovascular outcomes, inconsistencies between randomized and observational evidence, and the absence of dedicated cardiovascular outcome trials—were highlighted to identify priorities for future research.

As a narrative review, this work did not follow the Preferred Reporting Items for Systematic Reviews and Meta-Analyses (PRISMA) reporting framework, and no formal risk-of-bias assessment or quantitative meta-analysis was performed. Although predefined search procedures and explicit eligibility criteria were applied to improve transparency and reproducibility, considerable heterogeneity in study design, patient populations, baseline cardiovascular risk, JAK inhibitor selectivity, comparator therapies, follow-up duration, cardiovascular outcome definitions, and analytical methodology precluded formal quantitative evidence synthesis. Furthermore, restriction to English-language publications may have introduced language bias. Nevertheless, a narrative review was considered the most appropriate methodological approach for integrating mechanistic, clinical, regulatory, and real-world evidence into a comprehensive framework for understanding cardiovascular safety and supporting individualized clinical decision-making regarding JAKi therapy in RA.

## 3 Mechanistic considerations

### 3.1 The JAK-STAT pathway and vascular biology

The JAK-STAT pathway is an evolutionarily conserved signaling system that transmits extracellular cytokine, growth factor, and interferon signals to the nucleus and regulates gene transcription (Stark et al., 2018; Philips et al., 2022). It plays major roles in immune responses, hematopoiesis, cell survival, proliferation, differentiation, and inflammation (Xue et al., 2023). Additionally, it mediates signaling for over fifty cytokines and associated mediators, making it highly relevant to both vascular biology and chronic inflammatory diseases (O'Shea et al., 2013; Morris et al., 2018).

**FIGURE 2:**
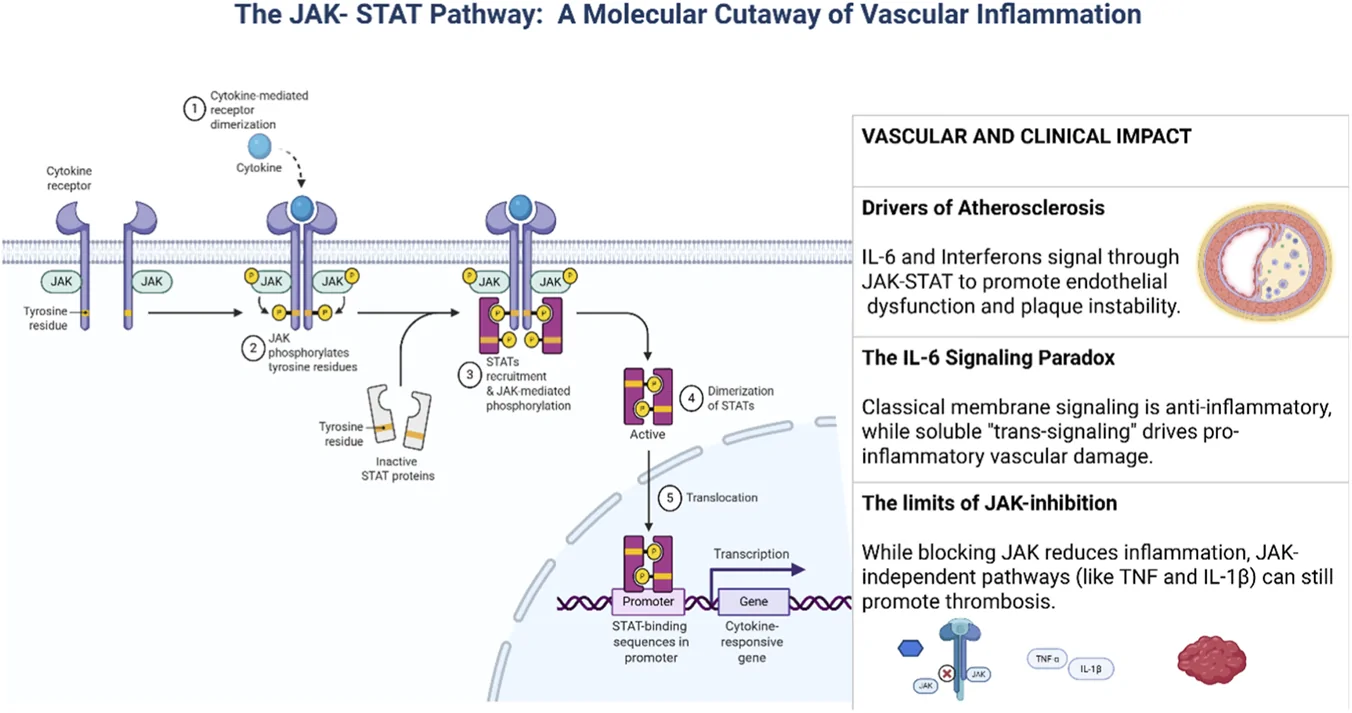
The JAK–STAT pathway: A molecular cutaway of vascular inflammation. Created in BioRender. Vučković, M (2026) https://BioRender.com/ahhapfh. Abbreviations: JAK, Janus kinase; STAT, signal transducer and activator of transcription; IL-6, interleukin-6; TNF, tumor necrosis factor; IL-1β, interleukin-1 beta.

The system consists of seven STAT proteins and four JAK kinases (JAK1, JAK2, JAK3, and TYK2) (Stark et al., 2018). Following ligand binding, receptor-associated JAKs recruit STAT proteins, phosphorylate receptor tyrosine residues, dimerize them, induce nuclear translocation, and activate transcription (Heim, 1996). This signaling is not uniform across tissues. Different receptors use different JAK combinations, and the relative abundance of JAKs within the cell may affect JAK-receptor binding (Zoler et al., 2024). This explains the varying vascular effects of pathway blockade and cytokine pleiotropy (Figure 2).

This is important in atherosclerosis. Cytokines that signal through JAK-STAT regulate endothelial activation, leukocyte recruitment, vascular inflammation, and plaque behavior. Among them, IL-6 is especially relevant. Endothelial dysfunction, monocyte trafficking, acute-phase activation, vascular inflammation, coagulation, and fibrosis are all facilitated by IL-6 (Preda et al., 2026). The association between persistent IL-6 activity and atherothrombotic risk supports the idea that JAK-dependent IL-6 suppression may have anti-atherogenic effects.

At the same time, IL-6 biology is more complex than a simple harmful signal. It has dual effects depending on the signaling mode. Classical signaling through the membrane-bound IL-6 receptor may exhibit anti-inflammatory properties, whereas trans-signaling through the soluble IL-6 receptor is more closely associated with pro-inflammatory and pro-atherogenic effects (Zhao and Mallat, 2019). Experimental and genetic evidence support this distinction. This suggests that blocking JAK may beneficially alter some IL-6-driven vascular pathways, but not in a fully predictable way.

Interferons add another layer. Type I interferons cause endothelial dysfunction, promote macrophage adhesion to endothelial cells, foam cell formation, and plaque development (Boshuizen and de Winther, 2015). IFN-γ also promotes macrophage and T-cell recruitment, foam cell formation, and plaque instability (Vuong et al., 2022). These findings support the hypothesis that suppression of JAK-mediated interferon signaling may mitigate vascular inflammation. However, interferons also participate in immune surveillance and host defense, so their inhibition is not automatically protective.

SOCS proteins, PIAS proteins, phosphatases, receptor endocytosis, and multiple “non-canonical” functions – including those of JAK in a kinase-independent manner and STAT without phosphorylation – all serve to tightly regulate the JAK-STAT pathway (Agashe et al., 2022; Mohr et al., 2012). Thus, the JAK-STAT pathway is much more than just an inflammatory switch; it is a well-organized network. Inhibiting this pathway could greatly decrease the detrimental effects of inflammation; however, it could also negatively affect the adaptive capabilities of the vascular system and the blood cell lineages resulting from hematopoiesis.

This explains why JAK inhibition can plausibly generate both benefit and risk. Experimental studies suggest anti-atherogenic effects, including reduced endothelial activation, reduced pro-thrombotic signaling, and reduced atherosclerosis in selected models (Tang et al., 2020; Beckman et al., 2023). Yet JAKis do not block all relevant inflammatory pathways equally. TNF, IL-17, and IL-1β remain active outside direct JAK control, and these pathways can still promote endothelial dysfunction and thrombosis (Zavoriti and Miossec, 2025). Pharmacovigilance signals for thromboembolic and cardiovascular events further support the need for caution (Zhong et al., 2025).

Overall, the vascular biology involved in the effects of JAKis suggests there may be a class effect, since all JAKis share the same signaling architecture. However, due to differences in the selectivity of JAKis for their respective kinases, the regulation of cytokines, and the different interactions of JAKis with thrombo-inflammatory biology, one cannot automatically assume a similar cardiovascular effect for all JAKis. Therefore, this pathway offers a biological rationale for the possibility of cardiovascular risk, but does not provide conclusive evidence of a common cardiovascular hazard for all JAKis.

### 3.2 Lipids and thrombosis

One of the most consistent biological effects of JAKis is an increase in blood lipid levels. Trials and meta-analyses have shown that these drugs cause an increase in both low-density lipoprotein cholesterol (LDL-C) and high-density lipoprotein cholesterol (HDL-C) within the first few weeks to months of treatment, followed by a period of stabilization (Isufi et al., 2026; Li et al., 2022; Qiu et al., 2019). The magnitude of change varies among compounds. TOFA has produced the largest increase in LDL-C compared to decernotinib, UPA, and baricitinib (Isufi et al., 2026). Additionally, the ratio of LDL to HDL has often remained essentially unchanged (Makris et al., 2022; Akar et al., 2026). This may indicate that these agents are not simply shifting lipids into pro-atherogenic forms, but are recalibrating the entire lipid metabolism system.

The results of these studies should also be interpreted in the context of the “lipid paradox” related to inflammation. For example, in inflammatory diseases such as RA, active joint inflammation is associated with lower cholesterol levels despite an increased risk for cardiovascular disease (Pérez-Baos et al., 2017; McInnes and Schett, 2017). This does not indicate that lipids are providing protection from vascular disease, but rather reflects disrupted lipid metabolism. The anti-atherogenic qualities of HDL are decreased during inflammation because HDL is modified by acute phase proteins, leading to impaired reverse cholesterol transport (Pérez-Baos et al., 2017; McInnes and Schett, 2017).

This promotes lipid retention within macrophages. Inhibition of JAK may partly reverse this accumulation. It has been proposed that increasing levels of JAK may lead to increased levels of LXRα and ATP-binding cassette transporter A1 (ABCA1), thereby improving reverse cholesterol transport (Pérez-Baos et al., 2017). Therefore, lipids that increase because of treatment could be a sign of normalized metabolism rather than direct vascular damage in these patients. Still, concern remains for patients with high baseline atherosclerotic risk. Routine lipid monitoring, therefore, remains necessary (Zhang et al., 2026).

The concept of thrombus formation introduces an additional mechanistic dimension. JAK2 is a central component of hematopoietic signaling and is essential for the growth of megakaryocytes and the production of platelets induced by thrombopoietin. MPL, the receptor for thrombopoietin, signals through JAK2. JAK2 mediates receptor stability and influences downstream pathways relevant to megakaryocyte differentiation (Besancenot et al., 2014). This is important when considering whether broader JAK inhibition may have different thrombo-inflammatory implications compared to more selective JAK1 inhibition. Thrombosis is characterized by gain-of-function mutations involving JAK2, such as the JAK2V617F mutation. This mutation results in persistent activation of the JAK-STAT signaling pathway, increased megakaryocyte production, and a strong association with arterial and venous thrombosis (Tefferi et al., 2025; Hobbs et al., 2013). Pharmacological inhibition of JAK cannot replicate the effects of a JAK2-activating mutation. However, the findings suggest that JAK2-related biological pathways are closely correlated with risk factors for thrombosis.

This creates a plausible mechanistic divergence between compounds. TOFA exhibits broader inhibition across a wide range of JAK pairings because it inhibits both JAK1 and JAK3, as well as influencing JAK2 signaling pathways. Baricitinib has greater inhibitory activity on the JAK1/JAK2 pathways than on the JAK1 and JAK3 pathways; therefore, it was developed as a JAK1-selective inhibitor (Traves et al., 2021; Virtanen et al., 2023). In theory, increased selectivity for JAK1 may preserve JAK2-dependent hematopoietic signaling pathways associated with erythropoietin, thrombopoietin, and GM-CSF (granulocyte-macrophage colony-stimulating factor) (Traves et al., 2021; Virtanen et al., 2023). However, ORAL Surveillance identified increased MACE risk with TOFA in patients with RA aged 50 years or older who had at least one additional cardiovascular risk factor (Ytterberg et al., 2022; Shah et al., 2024). Selectivity alone is therefore unlikely to explain the full clinical picture.

The cardiovascular implications remain unsettled. Shorter trials have not consistently shown a significant increase in cardiovascular events across JAKis overall. However, ORAL Surveillance identified increased MACE risk with TOFA 10 mg twice daily in RA patients aged 50 years or older with cardiovascular risk factors (Ytterberg et al., 2022; Shah et al., 2024). Whether this finding extends to more selective JAK1 inhibitors or to lower-risk populations remains unclear (Clarke et al., 2021; Núñez et al., 2023). Proposed mechanisms include enhanced thromboxane A2 biosynthesis in platelets and incomplete control of TNF- and IL-17-driven pro-coagulant endothelial signaling (Zavoriti and Miossec, 2025; Alabbasi et al., 2025).

In conclusion, the relationship between lipids and thrombosis is mechanistically coherent but clinically unresolved. JAK inhibition affects both lipid metabolism and thrombo-inflammatory biology. However, the net cardiovascular effect likely depends on the balance between reduced systemic inflammation, restoration of lipid trafficking, degree of JAK2 pathway engagement, drug selectivity, dose, and baseline vascular risk. Lipid changes and thrombosis biology should therefore be considered as interacting components of a broader cardiovascular risk framework, rather than as isolated warning signals.

## 4 Evidence from randomized controlled trials

### 4.1 Tofacitinib (TOFA)

#### 4.1.1 ORAL surveillance findings

The ORAL Surveillance was a randomized, post-authorization, noninferiority, phase 3b–4 safety trial that assessed the safety and efficacy of TOFA versus a TNFi in patients with RA aged 50 years or older with at least one additional cardiovascular (CV) risk factor (Ytterberg et al., 2022). This 4-year study focused on safety endpoints: MACE and malignancies, excluding non-melanoma skin cancer (NMSC) as primary endpoints (Charles-Schoeman et al., 2023). Patients with active RA despite methotrexate (MTX) treatment were randomly allocated in a 1:1:1 ratio to receive TOFA at doses of 5 mg or 10 mg twice daily, or a subcutaneous TNFi, specifically etanercept or ADA (Singh, 2022).

The size and duration of this study allowed for the assessment of a wide range of adverse events, including those with low incidence and/or extended latency, within a single cohort of patients in a prospective, randomized framework. In this cohort with elevated CV risk, the incidence of MACE and malignancies was higher with TOFA compared to TNFis, failing to meet noninferiority criteria (Ytterberg et al., 2022).

The noninferiority criterion for MACE was not met (Ytterberg et al., 2022). To elaborate, the trial prospectively specified that noninferiority would be demonstrated only if the upper bound of the two-sided 95% confidence interval for the hazard ratio was <1.8. This prespecified margin was agreed with regulatory authorities to exclude an unacceptable increase in cardiovascular risk with tofacitinib relative to TNFis. Although the estimated hazard ratio for MACE was 1.33, the upper limit of the 95% confidence interval (1.94) exceeded the prespecified margin; therefore, noninferiority could not be demonstrated (Ytterberg et al., 2022).

After 4 years, TOFA showed inferior outcomes compared to TNFi, with hazard ratios of 1.33 (95% CI 0.91–1.94) for MACE. The authors projected that over a 5-year therapy period, 113 patients would need to be treated with TOFA instead of a TNFi to result in one additional MACE (Ytterberg et al., 2022).

The observational, non-randomized STAR-RA study, which compared TOFA with TNFi, found a similar 1.24-fold (95% CI 0.90, 1.69) incidence of MACE in a cohort designed to replicate the high-risk ORAL Surveillance population (Khosrow-Khavar et al., 2022).

#### 4.1.2 Dose-dependent signal

In February 2019, the TOFA dose of 10 mg twice daily was reduced to 5 mg twice daily after the Data Safety Monitoring Board observed a higher incidence of pulmonary embolism (PTE) in patients receiving the 10 mg dose compared to TNFi, as well as increased overall mortality associated with the 10 mg dose relative to the 5 mg dose and TNFi (Charles-Schoeman et al., 2023).

#### 4.1.3 High-risk population characteristics

ORAL Surveillance included a CV risk-enriched cohort of patients with RA aged 50 years or older, with at least one additional CV risk factor: current smoking, hypertension, high-density lipoprotein cholesterol (HDL-c) levels below 40 mg/dL, diabetes mellitus, a family history of premature coronary heart disease (CHD), RA-associated extra-articular disease, and/or a history of coronary artery disease (CAD) (Ytterberg et al., 2022; Karpouzas et al., 2023).

The study included patients with and without a history of atherosclerotic CV disease (ASCVD) (Charles-Schoeman et al., 2023). A history of ASCVD was defined as a history of CAD, cerebrovascular disease (CeVD), or peripheral artery disease (PAD). A history of CAD was required for eligibility in ORAL Surveillance, defined as having at least one of the following: myocardial infarction (MI), unstable angina, stable angina pectoris, coronary artery procedures, or other forms of CHD. A history of CeVD (including ischemic stroke and transient ischemic attack) and PAD was identified in patients’ general medical history using the recommended terms of the Medical Dictionary for Regulatory Activities (Ytterberg et al., 2022). Nearly 15% of participants in ORAL Surveillance had a history of ASCVD (Charles-Schoeman et al., 2023).

#### 4.1.4 Statistical interpretation

The interpretation of ORAL Surveillance data may not be as straightforward as it initially appears. RA disease activity is recognized as being associated with an increased risk of MACE (Choy et al., 2014). Patients with RA have an estimated 70% higher risk of CV disease compared to the general population (Taylor et al., 2019).

The observed higher risk of MACE with TOFA than with TNFi in ORAL Surveillance may partly reflect a relative cardiovascular benefit of TNF inhibition, although the absence of a placebo comparator precludes separation of direct JAKi harm from comparator benefit (Ytterberg et al., 2022; Harrington et al., 2023). Accordingly, in older patients or those with established ASCVD or multiple cardiovascular risk factors, a TNFi or another non-JAK biologic may be preferable when clinically suitable, consistent with current regulatory risk-minimization recommendations (EMA, 2022; Smolen et al., 2022). The exploratory nature and lack of statistical evidence (i.e., nominally significant p-values) regarding the interaction between therapy and history of ASCVD limit conclusions about this subgroup analysis. The distribution of subgroups was uneven, with 14.7% having a history of ASCVD and 85.3% without such a history. In the ASCVD cohort, across treatment groups, there were very few patients (n = 204–222) and instances of MACE (n = 9–17). As a result, IRs and HRs should be considered statistically uncertain, as indicated by the broader 95% confidence intervals, and should be interpreted with caution. This analysis highlights the need for additional data on the risk of MACE associated with TOFA compared to other advanced RA therapies in patients with elevated CV risk but no prior history of ASCVD (Charles-Schoeman et al., 2023).

#### 4.1.5 Study limitations

The trial’s limitations include its open-label design, high rates of treatment discontinuation, lack of additional control groups, and the use of ADA in North America, while etanercept is used elsewhere globally. Other endpoints were not evaluated (Karpouzas et al., 2023). This study was not designed to compare the risk of venous thromboembolism (VTE) among therapies. The specificity of the risks to this patient population and to TOFA, compared to other JAKis, remains unclear, as does the relative risk between ADA and etanercept. The analyses were not adjusted for multiple comparisons (Ytterberg et al., 2022).

### 4.2 Baricitinib

#### 4.2.1 Integrated safety data

The safety profile of baricitinib for RA treatment is based on clinical trial data covering more than 14,000 patient-years of exposure (Taylor et al., 2022). In the 24-week placebo-controlled phase of the baricitinib RA clinical program, a numerical VTE imbalance was observed between baricitinib 4 mg (6 of 997 patients) and placebo (0 of 1,070 patients), suggesting a potential early thrombotic signal (Karpouzas et al., 2023). However, the short comparison period and limited accumulated patient-time constrained the assessment of rare outcomes such as MACE and VTE (Taylor et al., 2022). A multi-database study evaluated the safety of baricitinib compared with TNFi in routine care, focusing on VTE, MACE, and serious infections across 14 post-marketing data sources in Europe, the United States, and Japan (Salinas et al., 2023). Baricitinib was associated with a significantly increased risk of VTE versus TNFi (IRR 1.51, 95% CI 1.10–2.08), whereas the incidence-rate difference was 0.26 (95% CI −0.04 to 0.57) per 100 patient-years (Salinas et al., 2023). MACE and serious infections were numerically more frequent with baricitinib, but neither difference was statistically significant (Salinas et al., 2023). The IRs of VTE, MACE, and serious infections in patients treated with baricitinib, derived from the clinical development program and other RA populations from various external sources, were numerically similar; however, no statistical comparison was performed (Taylor et al., 2022).

#### 4.2.2 MACE incidence

According to the final integrated analysis of the baricitinib clinical development program (>14,000 patient-years; median exposure, 1,683 days; maximum exposure, 3,405 days), the incidence rate of MACE—defined as myocardial infarction, stroke, or cardiovascular death—remained stable at 0.51 (95% CI 0.40–0.64) per 100 patient-years during a median treatment duration of 4.6 years (maximum, 9.3 years) (Taylor et al., 2022). No clear dose-related difference was observed between baricitinib 2 mg and 4 mg, although the number of adjudicated events was limited (Taylor et al., 2022).

Salinas et al. reported a numerically higher, but not statistically significant, incidence of MACE with baricitinib than with TNFi (IRR 1.54, 95% CI 0.93–2.54; IRD 0.22, 95% CI −0.07 to 0.52 per 100 patient-years) (Salinas et al., 2023). Estimates differed across the two largest contributing data sources: ARTIS IRR 0.94 (95% CI 0.45–1.96) and SNDS IRR 2.33 (95% CI 1.15–4.74) (Salinas et al., 2023). This heterogeneity may reflect differences in channeling, disease refractoriness, comorbidity burden, and residual confounding across databases (Salinas et al., 2023).

Across the long-term baricitinib program, MACE incidence remained low and did not increase with longer exposure (Taylor et al., 2022; Genovese et al., 2020). However, indirect comparisons with TNFi and other JAKi development programs should not be interpreted as evidence of equivalence because the populations, follow-up durations, and event-ascertainment methods differed (Taylor et al., 2022; Genovese et al., 2020). Available data also did not demonstrate a clear dose-related difference between baricitinib 2 mg and 4 mg, but event counts were limited (Taylor et al., 2022).

#### 4.2.3 Venous thromboembolism (VTE) considerations.

Similar to CV disease in RA, studies have shown that patients with RA have an increased risk of VTE compared to the healthy population (Molander et al., 2021). The prevailing assumption is that strict disease management should reduce the risk of VTE.

Before ORAL Surveillance, a VTE imbalance emerged during the placebo-controlled phase of baricitinib development, particularly with the 4 mg dose (Genovese et al., 2020; Desai et al., 2021). This raised concern that thrombotic risk might not be confined to TOFA. In longer-term analyses, however, VTE incidence did not continue to increase, and randomized-trial meta-analyses have not consistently demonstrated excess VTE across JAKis (Genovese et al., 2020; Desai et al., 2021; Yates et al., 2021). These findings support continued caution but do not establish a uniform class effect. A meta-analysis of 42 randomized controlled trials found no increased risk of VTE in RA patients on JAKis compared to those on PBO (Yates et al., 2021). As noted, RA itself increases the risk of VTE, especially during periods of active disease. This meta-analysis may help alleviate concerns about the inherent pro-thrombotic potential of the JAKi class.

#### 4.2.4 Comparisons with TNF inhibitors

The RA-BEAM trial was a global, phase 3, double-blind study that included patients with moderately to severely active RA, using both PBO and active controls (Taylor et al., 2017). The trial aimed to assess changes in disease activity, structural integrity, and PROs (including physical function), as well as the safety and adverse effect profile of a daily 4 mg oral baricitinib regimen in patients with an inadequate response to MTX. Comparisons were made with PBO and the TNFi, ADA, a biologic disease-modifying antirheumatic drugs (bDMARD) for patients with moderately to severely active RA. Baricitinib demonstrated noninferiority to ADA at week 12 for the American College of Rheumatology 20% improvement response (ACR20), with a noninferiority margin of 12%. The statistical analysis plan showed that baricitinib was considerably superior to ADA (p = 0.01). Additionally, baricitinib was superior to ADA in mean change in Disease Activity Score for 28 joints using high-sensitivity C-reactive protein (DAS28-CRP) at week 12. Baricitinib was associated with improvements in signs and symptoms measured by DAS28-CRP and the Simplified Disease Activity Index (SDAI), physical function assessed by the Health Assessment Questionnaire–Disability Index (HAQ-DI), PROs, and progression of structural joint damage compared to PBO, as well as improvements in ACR20 response and DAS28-CRP compared to ADA (Taylor et al., 2017).

#### 4.2.5 Lack of a clear excess MACE signal but limited statistical power

To date, the evidence does not show a consistent or statistically significant increase in MACE compared to PBO or active comparators (Taylor et al., 2022; Dougados et al., 2017). The incidence rates of MACE observed in baricitinib clinical development programs have generally been low and similar to those expected in RA populations with comparable baseline cardiovascular risk profiles (Taylor et al., 2022). However, interpretation of these results is limited by insufficient statistical power (Ytterberg et al., 2022; Taylor et al., 2022).

Most randomized controlled trials (RCTs) of baricitinib were not specifically designed or adequately powered to detect differences in relatively rare safety outcomes such as MACE (Ytterberg et al., 2022; Taylor et al., 2022). As a result, the total number of adjudicated CV events remains small, leading to wide confidence intervals for risk estimates and making it difficult to rule out a small increase in risk (Taylor et al., 2022). Additionally, trial populations often do not adequately represent patients at the highest CV risk, potentially limiting the detection of safety signals (Ytterberg et al., 2022).

Long-term extension data and integrated safety analyses have provided additional follow-up, but are limited by factors such as the absence of comparator arms and the potential for selection bias (Taylor et al., 2022). Although observational and real-world studies may offer further insights, these data are still emerging and may be affected by confounding factors (EMA, 2023).

Given the broader JAKi class, especially after the results of the ORAL Surveillance trial with TOFA, regulatory agencies have emphasized the need for careful interpretation of CV safety among these agents (Khosrow-Khavar et al., 2022; Karpouzas et al., 2023). Baricitinib has not shown a clear excess MACE signal, but current evidence does not allow for definitive conclusions about cardiovascular risk. More studies with more power and continued pharmacovigilance are still needed (Ytterberg et al., 2022; Karpouzas et al., 2023).

### 4.3 Upadacitinib

#### 4.3.1 SELECT clinical trial program

The SELECT Phase III program in RA included six global phase III studies (Burmester et al., 2018; Genovese et al., 2018). Positive clinical efficacy data and an acceptable benefit-risk profile led to the approval of UPA 15 mg once daily by the United States FDA and the European Medicines Agency EMA for the treatment of moderate to severe RA, either as monotherapy or in combination with conventional synthetic disease-modifying antirheumatic drugs (csDMARDs) for patients who have not responded to MTX (Smolen et al., 2019). Two studies also demonstrated the non-inferiority or superiority of UPA compared to another bDMARD.

UPA monotherapy showed statistically significant improvements in clinical and functional outcomes compared to continued MTX in the SELECT MONOTHERAPY study. The SELECT EARLY study evaluated the clinical efficacy of UPA monotherapy *versus* MTX monotherapy in MTX-naïve patients with moderate to severe active RA (van Vollenhoven et al., 2018). The SELECT NEXT study was a randomized, double-blind, PBO-controlled trial with 661 patients with active RA who had an inadequate response to csDMARDs (Burmester et al., 2018).

SELECT BEYOND was a randomized controlled trial that included 499 patients with RA who were previously inadequate responders or intolerant to bDMARDs (Genovese et al., 2018). SELECT-CHOICE was a phase 3, double-blind, head-to-head study in patients with insufficient response to bDMARDs, evaluating the efficacy and safety of UPA *versus* abatacept (ABA), both administered alongside stable background csDMARDs (Rubbert-Roth et al., 2020).

A notable strength of the SELECT studies was the statistical analysis used in data evaluation, including adjustments for multiplicity and non-responder imputation.

#### 4.3.2 Head-to-head comparison with adalimumab

The SELECT COMPARE study assessed the efficacy of UPA relative to PBO or ADA in patients with an inadequate response to MTX. Included patients had active disease, with a mean RA duration since diagnosis of 8 years. Participants were randomized to receive UPA 15 mg, PBO, or ADA (40 mg biweekly) while maintaining a consistent background dose of MTX. This trial was designed and powered to demonstrate superiority over PBO and to assess the noninferiority of UPA compared to ADA, followed by an evaluation of superiority, as measured both clinically and functionally. At week 12, both primary endpoints were met in patients receiving UPA compared to those receiving PBO (p ≤ 0.001). UPA demonstrated superiority over ADA in terms of the ACR50 response rate, changes in pain severity score, and changes in the HAQ DI. At week 26, more patients receiving UPA than those receiving PBO or ADA achieved low disease activity or remission (p < 0.001) (Fleischmann et al., 2019). The SELECT COMPARE study, conducted over 48 weeks, showed that responses to UPA treatment were sustained throughout the study and remained consistently superior to those with ADA. Patients who did not achieve a 20% improvement in tender joint count with ADA or UPA were switched to the alternative medication. This is the first data to evaluate the response in patients who did not respond to the JAKi and were switched to TNFi.

#### 4.3.3 Observed MACE rates

The efficacy and safety of the JAKi UPA have been evaluated in a diverse cohort of patients with RA through the SELECT clinical trial program. In the SELECT EARLY trial, the rates of adjudicated MACE, adjudicated VTE, and gastrointestinal perforations were similar between the UPA and PBO groups (van Vollenhoven et al., 2020).

In a *post hoc* analysis of the SELECT program, adjudicated MACE rates were low and broadly similar across UPA 15 mg, ADA, and MTX, both in the overall population and among patients with elevated CV risk (Fleischmann et al., 2023). In the higher-risk SELECT-COMPARE subgroup, UPA was not associated with increased MACE relative to ADA (HR 0.63, 95% CI 0.13–3.07), although the wide confidence interval indicates substantial imprecision (Fleischmann et al., 2023). Older age, smoking history, and prior CV events were associated with higher event rates irrespective of treatment (Fleischmann et al., 2023). In the long-term extension of the phase 3 SELECT-CHOICE study, adjudicated MACE and VTE remained uncommon during exposure to UPA 15 mg. Because event counts were small and the extension phase lacked a concurrent comparator, these findings are reassuring but cannot exclude a modest increase in risk (Rubbert-Roth et al., 2024).

#### 4.3.4 Interpretation caveats (younger populations, shorter follow-up)

Several important interpretation caveats should be considered when evaluating the safety of UPA. First, the clinical trial populations for UPA have generally consisted of younger patients with lower baseline CV risk compared to real-world RA populations, especially those in post-marketing safety studies of other JAKis (Ytterberg et al., 2022; Cohen et al., 2021). This demographic profile may limit the generalizability of the observed CV safety outcomes and reduce the likelihood of detecting potential risk signals. Second, the follow-up period in RCTs and even long-term extension studies remains insufficient to assess rare outcomes such as MACE (Cohen et al., 2021). Although integrated analyses have increased the total number of patient-years of exposure, the actual number of adjudicated CV events remains low, resulting in wide confidence intervals and low statistical precision (Cohen et al., 2021).

Additionally, cross-trial comparisons with studies such as the ORAL Surveillance trial are limited by differences in study design, patient selection, and risk enrichment strategies, further complicating interpretation (Ytterberg et al., 2022).

In summary, although current evidence does not indicate a definitive increased risk of MACE associated with UPA, the relatively younger trial populations and shorter follow-up periods require careful interpretation. Continued pharmacovigilance and real-world evidence will be essential to more conclusively determine long-term cardiovascular safety (Cohen et al., 2021; EMA, 2023).

### 4.4 Filgotinib

#### 4.4.1 FINCH clinical trial program

The advancement of filgotinib included four phase III clinical trials within the FINCH program. The FINCH 1 study was a randomized, double-blind, PBO-controlled trial (Combe et al., 2019). Patients with active RA and an inadequate response to MTX were randomized to receive either oral filgotinib 200 mg or 100 mg daily, subcutaneous ADA 40 mg biweekly, or a matching PBO for up to 52 weeks. At week 24, patients who received PBO were re-randomized to filgotinib. Response rates were quantitatively similar between those administered filgotinib 100 mg and those receiving ADA. Filgotinib showed a favorable benefit-risk profile in MTX inadequate responders with active RA (Combe et al., 2020).

The FINCH 2 study evaluated the efficacy of filgotinib compared to PBO in patients with moderately to severely active RA who had an unsatisfactory response or intolerance to one or more prior bDMARDs (Genovese et al., 2019). Patients were randomly assigned to receive once-daily filgotinib 200 mg, filgotinib 100 mg, or PBO. Responses to the 200 mg dose were quantitatively superior to those of the 100 mg dose; however, no statistical analysis was conducted to assess a potential dose-response relationship.

The FINCH 3 trial included MTX-naive patients with active RA, randomized to receive oral filgotinib 200 mg once daily with MTX, filgotinib 100 mg with MTX, filgotinib 200 mg as monotherapy, or MTX at a dosage of ≤20 mg weekly (Westhovens et al., 2019). In the monotherapy group, a higher percentage of patients achieved ACR 50/70 with filgotinib 200 mg compared to MTX; however, the ACR 20 response was not substantially higher at week 24. The efficacy of filgotinib was maintained through week 52 (Westhovens et al., 2020).

The FINCH 4 study assesses the long-term safety and tolerability of filgotinib in people who have completed a parent study of filgotinib for RA (Buch et al., 2024)**.**

#### 4.4.2 Selectivity for JAK1

JAK1-selective agents have demonstrated significant efficacy in managing RA, consistent with findings from other pan-JAKis. The rapid onset of response is a distinctive feature of this class, as shown in the UPA/filgotinib trials. Head-to-head trials are needed to determine any differences in efficacy among JAKis. In the absence of such data, indirect comparisons using real-world data from observational registries are essential for understanding the potential advantages of JAK1-selective inhibition compared to pan-JAK inhibition (Biggioggero et al., 2019), as well as for meta-analyses (Pope et al., 2020).

#### 4.4.3 No strong MACE signal to date

Comprehensive evaluations of long-term integrated safety data from the DARWIN and FINCH studies indicate that the safety and tolerability profiles of filgotinib at 100 mg and 200 mg are largely comparable. Rates of MACE are low and similar across the different filgotinib dosages (Winthrop et al., 2022; Burmester et al., 2024). The event rates of MACE were minimal across dosage groups and remained consistent over time; the event rates (95% CI) per 100 patient-years of MACE were 0.5 (0.3–0.7) for the 100 mg group and 0.3 (0.2–0.5) for the 200 mg group. In the age subgroup analysis, the EAIR of MACE was higher in patients aged ≥65 years compared to their younger counterparts; the EAIRs (95% CI) per 100 patient-years for MACE were identical in patients aged ≥65 years receiving filgotinib 200 mg (1.0 [0.5 to 1.7]) or 100 mg (1.0 [0.5 to 1.9]). The event rates of MI were modest, with overlapping 95% confidence intervals between dosage groups (Burmester et al., 2024). The incidence of MACE was comparable across filgotinib dosages in the overall group.

#### 4.4.4 Limited long-term exposure

The assessment of filgotinib’s long-term safety, especially for rare events, is limited by shorter cumulative exposure than for several other JAKis (Combe et al., 2020; Genovese et al., 2019; Burmester et al., 2024). The FINCH clinical trial program has shorter follow-up and fewer total patient-years of exposure, resulting in less information on long-latency outcomes such as cardiovascular events and malignancy (Combe et al., 2020; Genovese et al., 2019). Integrated safety analyses have not revealed a definitive excess-risk signal; however, the limited number of adjudicated events reduces precision and restricts the ability to exclude a small increase in risk (Winthrop et al., 2022; Burmester et al., 2024). Filgotinib was not approved for rheumatoid arthritis in the United States: after an FDA Complete Response Letter, the sponsor announced in December 2020 that it would not pursue US approval for this indication (Gilead Sciences, 2020). Its use has therefore been concentrated mainly outside the United States, particularly in Europe and Japan, which may partly explain the thinner long-term US real-world evidence base compared with other JAKis (Gilead Sciences, 2020; EMA, 2023). Ongoing post-marketing surveillance and international real-world data remain essential for defining its long-term cardiovascular safety (EMA, 2023; Burmester et al., 2024).

## 5 Real-world evidence and clinical implications

### 5.1 Observational and registry data

While RCTs provide the most robust evidence on the cardiovascular safety of JAKis, their generalizability is often limited by highly selected patient populations and protocol-driven treatment conditions. In contrast, real-world evidence (RWE) offers insight into treatment effects across broader, older, and more comorbid populations encountered in routine practice. However, these data are inherently vulnerable to confounding by indication, channeling bias, outcome misclassification, and incomplete adjustment for baseline cardiovascular risk. Therefore, integrating findings from observational studies, registries, and pharmacovigilance databases is essential for a more comprehensive understanding of cardiovascular risk associated with JAKis (Hoisnard et al., 2022; Goldman et al., 2024a; Zhong et al., 2025; Khosrow-Khavar et al., 2022).

RWE complements RCTs in evaluating the association between JAKi and MACE, particularly because it captures patients often excluded from trials, including those with greater comorbidity burden and higher baseline cardiovascular risk. However, this broader clinical applicability comes with increased susceptibility to bias and residual confounding. Pharmacovigilance databases such as VigiBase and FAERS are useful for signal detection but cannot provide true incidence estimates and are prone to stimulated reporting, selective reporting, and channeling bias. Nonetheless, disproportionality analyses have suggested increased reporting of cardiovascular events with JAKis, with heterogeneity across specific agents and cardiovascular outcomes (Hoisnard et al., 2022; Goldman et al., 2024a; Zhong et al., 2025). Observational cohort and registry-based studies allow head-to-head comparisons between JAKis and bDMARDs, particularly TNFi, under routine care conditions. In the STAR-RA study, TOFA was not associated with a clear increase in cardiovascular outcomes in the overall real-world RA population, although risk estimates were numerically higher in patients with cardiovascular risk factors, aligning directionally with ORAL Surveillance. Similarly, a recent frailty-focused real-world comparative study suggested that baseline patient vulnerability and cardiovascular risk profile substantially influence observed safety signals with JAKi *versus* TNFi (Khosrow-Khavar et al., 2022; Ytterberg et al., 2022; Silvagni et al., 2025).

Importantly, newer multinational registry data from the JAK-pot collaboration did not show an increased incidence of MACE with JAKis compared with TNFi during the first 2 years of treatment in the general RA population, although absolute event rates were predictably higher in cardiovascular-enriched subsets. These findings suggest that excess cardiovascular risk may not be uniform across all treated patients, and that discrepancies between trials and observational studies may partly reflect differences in patient selection, comparator choice, and underlying cardiovascular risk enrichment. When interpreted alongside ORAL Surveillance and its *post hoc* analyses, the totality of evidence suggests that any excess cardiovascular hazard with JAK inhibition is most evident in patients with established atherosclerotic cardiovascular disease, advanced age, smoking history, or multiple baseline cardiovascular risk factors (Ytterberg et al., 2022; Aymon et al., 2025; Charles-Schoeman et al., 2023).

Regulatory authorities have therefore adopted a precautionary approach. Following review of randomized and observational data, the European Medicines Agency concluded that the identified risks should be considered relevant to JAKis used for chronic inflammatory disorders as a class, particularly in high-risk patients, and recommended preferential use of alternative treatments when suitable options exist in patients aged 65 years or older, current or past long-term smokers, and those with cardiovascular or malignancy risk factors. Overall, current RWE does not conclusively support either a uniform class effect or a purely molecule-specific risk. Rather, it supports a nuanced interpretation in which drug-specific properties, patient-level cardiovascular risk, and confounding by indication all likely contribute to the observed heterogeneity (Khosrow-Khavar et al., 2022; Ytterberg et al., 2022; Silvagni et al., 2025; Aymon et al., 2025; Charles-Schoeman et al., 2023; EMA, 2022).

Emerging evidence on JAKis and MACE has important implications for clinical decision-making. Current data suggest that cardiovascular risk associated with JAK inhibition is not uniform across all patients and is not yet proven to be identical across all molecules, leaving the question of class effect versus molecule-specific risk unresolved. Findings from ORAL Surveillance and subsequent *post hoc* analyses indicate that excess MACE risk is concentrated mainly in patients with established atherosclerotic cardiovascular disease or multiple baseline cardiovascular risk factors, rather than in all treated populations. Observational studies have produced more heterogeneous results, with some showing no excess risk in broader RA populations and others suggesting greater concern in high-risk subgroups (Khosrow-Khavar et al., 2022; Ytterberg et al., 2022; Silvagni et al., 2025; Aymon et al., 2025; Charles-Schoeman et al., 2023).

From a practical perspective, cardiovascular risk assessment should be performed before initiating JAKi therapy, especially in patients with RA, who already have elevated background cardiovascular risk due to systemic inflammation and conventional risk factors. Particular caution is warranted in individuals aged 65 years or older, current or former long-term smokers, and those with previous atherosclerotic cardiovascular disease or multiple cardiovascular risk factors, in line with regulatory recommendations. In such patients, the decision to prescribe a JAKi should be individualized and weighed against available biologic alternatives, disease severity, prior treatment response, and patient preferences (EMA, 2022; Smolen et al., 2022).

Potential differences in JAK selectivity raise the possibility of molecule-specific variability, although the strongest signal remains for TOFA, as it is supported by the most definitive randomized safety dataset. In contrast, available long-term integrated safety analyses for UPA have not demonstrated a clear excess of MACE relative to active comparators within clinical trial programs, although these data should not be interpreted as definitive proof of lower cardiovascular risk across all clinical settings. Moreover, experience with other targeted therapies shows that adverse lipid changes do not necessarily translate into worse cardiovascular outcomes. For example, large real-world cohort studies found no increase in MACE with tocilizumab compared with TNFi despite its effects on lipid parameters. This reinforces the concept that cardiovascular outcomes may differ according to the inflammatory pathway targeted and the risk profile of the treated population (Ytterberg et al., 2022; Charles-Schoeman et al., 2023; Burmester et al., 2025; Kim et al., 2017).

Overall, the available evidence supports a risk-stratified rather than uniformly restrictive approach to JAKi use. In patients at lower baseline cardiovascular risk, JAKi may remain appropriate when clinically indicated. In contrast, in patients at high cardiovascular risk, clinicians should carefully consider alternative biologic therapies, optimize modifiable cardiovascular risk factors, and maintain closer follow-up during treatment. Shared decision-making remains central, particularly when balancing the benefits of disease control against potential cardiovascular harm in individuals with complex risk profiles (EMA, 2022; Smolen et al., 2022).

### 5.2 Discordance between RCTs and real-world data

A consistent discrepancy exists between randomized RCTs and RWE regarding the cardiovascular safety of JAKi. Most notably, the ORAL Surveillance trial found a higher risk of MACE with TOFA than with TNFi among patients with RA aged 50 years or older who had at least one additional cardiovascular risk factor (Ytterberg et al., 2022). Additional *post hoc* analyses further suggested that the excess risk was concentrated particularly among patients with established ASCVD or a markedly elevated cardiovascular risk profile (Charles-Schoeman et al., 2023).

In contrast, observational studies, registry analyses, and large comparative meta-analyses have generally reported similar MACE rates with JAKi and biologic therapies and have not consistently reproduced the signal observed in ORAL Surveillance. For example, in the US STAR-RA claims-based study, TOFA was not associated with a significantly increased cardiovascular risk in the overall RA population, although point estimates were higher in the subgroup designed to emulate ORAL Surveillance (Khosrow-Khavar et al., 2022). Similarly, the French nationwide SNDS cohort found no excess risk of MACE or venous thromboembolism with JAKis versus ADA, including among higher-risk patients (Hoisnard et al., 2023). Data from the German RABBIT registry, Swedish routine-care analyses, a Korean nationwide cohort restricted to patients without baseline cardiovascular disease, and the Spanish BIOBADASER registry also did not confirm a clear increase in MACE versus TNFi or other DMARDs (Meissner et al., 2023; Bower et al., 2023; Song et al., 2023; Hernández-Cruz et al., 2024).

A recent systematic review and meta-analysis including more than 800,000 patients with immune-mediated inflammatory diseases also found no significant difference in MACE risk between JAKi and TNFi (HR, 0.91; 95% CI, 0.80–1.04), although event incidence was low overall and venous thromboembolism appeared modestly increased in RA (Solitano et al., 2025). Pooled analyses of randomized trials and network meta-analyses likewise have not shown a significant increase in MACE risk across JAKis as a class, although signals for all-cause mortality appear to be driven predominantly by TOFA (Wei et al., 2023). More recent trial-level meta-analyses also suggest that JAKis do not significantly increase MACE risk overall, despite continued concern regarding other adverse outcomes (Duan et al., 2026).

Several methodological and clinical factors may explain these discordances. First, differences in patient selection are critical. ORAL Surveillance intentionally enrolled older patients with RA and established cardiovascular risk enrichment, thereby maximizing event rates and improving statistical power for safety signal detection (Ytterberg et al., 2022; Charles-Schoeman et al., 2023). In contrast, real-world datasets usually include broader and more heterogeneous populations, many of whom are at substantially lower baseline cardiovascular risk. This likely lowered absolute event rates and possibly attenuated the ability to detect treatment-related signals (Hoisnard et al., 2023; Song et al., 2023; Hernández-Cruz et al., 2024).

Second, the choice of comparator substantially influences relative risk estimation. TNFis, which served as active comparators in ORAL Surveillance, may themselves exert cardioprotective effects through suppression of systemic inflammation (Barnabe et al., 2011). Therefore, the excess risk observed with TOFA may in part reflect a relative benefit of TNF inhibition rather than a uniformly direct proatherogenic effect of JAK inhibition. In the absence of PBO-controlled cardiovascular outcome trials, separating absolute drug harm from comparator benefit remains challenging.

Third, differences in study design and outcome ascertainment significantly contribute to the observed divergence. RCTs rely on prespecified definitions, systematic surveillance, and formal adjudication of cardiovascular events, thereby maximizing internal validity. In contrast, observational studies commonly depend on administrative coding or registry-based outcome capture, which, although often validated, may introduce misclassification and variability in event detection (Khosrow-Khavar et al., 2022; Meissner et al., 2023; Hoisnard et al., 2023; Bower et al., 2023; Song et al., 2023; Hernández-Cruz et al., 2024). In addition, exposure duration and follow-up may differ substantially across routine-care datasets, which can further limit the ability to detect delayed cardiovascular toxicity (Solitano et al., 2025).

Fourth, RWE is highly vulnerable to confounding by indication and other biases. In routine practice, JAKis are frequently prescribed later in the treatment pathway to older patients with more refractory disease, more prior biologic exposure, greater comorbidity burden, and higher intrinsic cardiovascular risk (Khosrow-Khavar et al., 2022; Meissner et al., 2023; Solitano et al., 2025). Similarly, after regulatory warnings and label changes, clinicians may preferentially avoid prescribing JAKis to patients perceived to be at the highest cardiovascular risk, which could artifactually lower observed event rates in more contemporary observational cohorts. This time-dependent effect may further widen the apparent discrepancy between RCTs and real-world studies.

From a methodological standpoint, these observations suggest that RCTs may amplify safety signals through deliberate risk enrichment and rigorous event adjudication, whereas real-world analyses may dilute such signals because of population heterogeneity, shorter or variable follow-up, and residual confounding. Rather than being mutually contradictory, these data likely offer complementary perspectives across different baseline risk strata. Taken together, the available evidence supports a context-dependent view of cardiovascular risk with JAKis, in which patient selection, comparator choice, drug-specific effects, and study design are all critical determinants of the observed signal.

## 6 Regulatory and guideline perspectives

Regulatory authorities have responded decisively to emerging cardiovascular safety signals associated with JAKis, particularly after the ORAL Surveillance trial. In 2021, the U.S. Food and FDA issued a boxed warning for TOFA, highlighting an increased risk of MACE, malignancies, and thrombosis; similar warnings were later extended to other approved JAKis (FDA, 2021). The EMA implemented parallel restrictions, including recommendations to limit JAKis use in patients aged 65 years or older, those with cardiovascular risk factors, or a history of malignancy (The Lancet Gastroenterology and Hepatology, 2023). Notably, these measures extended beyond TOFA–the molecule directly implicated in pivotal trials–to the entire class, despite limited direct evidence for baricitinib, UPA, or filgotinib (FDA, 2021; The Lancet Gastroenterology and Hepatology, 2023). Subsequently, professional societies have also issued guidance, recommending cardiovascular risk stratification and consideration of alternative biologics in high-risk patients (Table 1) (Nash et al., 2023; Konzett et al., 2025).

**TABLE 1:** Regulatory actions and guideline recommendations for JAK inhibitors in relation to cardiovascular safety.

Agency/Organization	Year/Action	Target	Key warning/Restriction	Notes/Evidence basis
FDA	2021	Tofacitinib	Boxed warning: Increased risk of MACE, malignancies, thrombosis	Triggered by ORAL surveillance trial (high CV-risk RA population)
FDA	2021–2022	Baricitinib, upadacitinib	Warnings extended to entire JAK class	Precautionary; limited direct CV outcome data
EMA	2021–2022	All approved JAK inhibitors	Restrict use in ≥65 years, CV risk factors, history of malignancy	Safety extrapolation; class-wide approach despite limited molecule-specific data
Rheumatology societies (ACR/EULAR)	2022–Ongoing updates	All JAK inhibitors	Recommend CV risk stratification; consider alternative biologics in high-risk patients	Reflects both regulatory guidance and clinical experience

Caption: CV, cardiovascular; MACE, major adverse cardiovascular events; FDA–U.S., food and drug administration; EMA, European Medicines Agency; RA, rheumatoid arthritis; JAK, Janus kinase; ACR, American College of Rheumatology; EULAR, European Alliance of Associations for Rheumatology.

The class-wide approach has generated debate within the rheumatology community, highlighting the tension between regulatory caution and molecule-specific evidence. While biologically plausible, the assumption of a universal class effect remains unproven, and the broad warnings may inadvertently influence therapeutic decision-making, especially in patients without elevated cardiovascular risk. Observational data indicate a measurable shift in prescribing patterns, with clinicians increasingly favoring TNFis or other biologics over JAKis, even when the latter might provide superior disease control (Tomelleri et al., 2024).

These regulatory perspectives emphasize the critical role of risk stratification and individualized therapy in clinical practice. They also highlight the need for ongoing post-marketing surveillance, dedicated cardiovascular outcome trials, and refined guidance that distinguishes molecule-specific risk from potential class effects. By balancing caution with evidence-based nuance, regulators aim to protect patients while preserving access to this transformative therapeutic class for inflammatory diseases. The following details and concepts will be discussed further in the upcoming paragraphs.

## 7 Class effect or molecule-specific risk?

### 7.1 Arguments supporting a class effect

Several observations support the interpretation that the cardiovascular safety signal associated with JAKis may, at least in part, reflect a class effect. This does not imply that every agent carries the same absolute risk; rather, it suggests that drugs sharing the same central signaling target may also share a common biological susceptibility to adverse cardiovascular outcomes (Figure 1).

This interpretation is strongly grounded in shared pharmacologic target engagement. All approved JAKis inhibit the JAK-STAT pathway, but each has different selectivity for blocks within this pathway. This is important because the JAK-STAT pathway influences not only the inflammatory process but also regulates endothelial function, thrombo-inflammatory balance, hematopoiesis, and lipid homeostasis (Shah et al., 2024; Baldini et al., 2021). The possibility of a class effect is therefore biologically plausible, since the same signaling architecture is perturbed across the entire drug category.

Regulatory practices also provide information supporting this conclusion. Following the ORAL Surveillance findings, which showed that TOFA was inferior to TNFis for MACE and VTE in RA patients aged 50 years or older with cardiovascular risk factors, both the FDA and EMA have adopted a class-wide risk management approach (Misra et al., 2023; Alabbasi et al., 2025). Warnings have been expanded not only to include TOFA but also other JAKis (e.g., baricitinib and UPA) based on their shared mechanism, and there is insufficient evidence to conclude that the other JAKis do not share a similar risk of toxicity with TOFA (Alabbasi et al., 2025; Zavoriti and Miossec, 2025). Such regulatory extrapolation does not prove identical toxicity across compounds, but it clearly reflects the judgment that common target biology justifies class-wide caution.

Multiple sources of pharmacovigilance data continue to support the conclusion of a class effect, along with recurrent cardiovascular signals associated with the use of various agents. A comprehensive global post-marketing study involving 75,407 patients with RA treated with different JAKis demonstrated that JAKis (as a class), compared to bDMARDs, were associated with an increased frequency of venous thromboembolic events, strokes, ischemic heart disease, peripheral edema, and tachyarrhythmias. The authors specifically indicated that these adverse events were reported for all JAKis assessed, supporting the hypothesis of an overall class effect (Goldman et al., 2024). In a separate analysis by the FDA’s Adverse Event Reporting System (FAERS), significant cardiovascular signals were also observed for baricitinib, TOFA, and UPA compared with TNFis (Zhong et al., 2025). However, the specific pattern of these signals varies between agents. While this similarity does not necessarily mean that the risks are identical regardless of which molecule is used, it adds to the complexity of determining whether these cardiovascular concerns are specific to one compound.

Product labeling also reflects this pattern, and the current JAKis approved for RA include warnings for MACE and thrombotic events (Goldman et al., 2024; Zhong et al., 2025). These warnings include similar risk minimization strategies, such as preferring lower doses of JAKis and avoiding or limiting their use in older patients, smokers, and patients with cardiovascular risk factors (Baldini et al., 2021; Alabbasi et al., 2025). The convergence in labeling language and prescribing precautions provides practical support for the idea that JAKis have a shared class vulnerability.

Mechanistic considerations further support this interpretation. Across all classes of JAKis, changes in lipid metabolism occur through increases in both LDL and HDL cholesterol levels. Additionally, there are likely effects on platelet and endothelial cell activation that promote thrombotic events (for example, through increased thromboxane A2 production) and potentially incomplete modulation of pro-coagulation mechanisms driven by TNF and IL-17 (Zavoriti and Miossec, 2025; David et al., 2026). These downstream effects may not be identical for all compounds, but they support the concept of a shared biological vulnerability rooted in JAK-STAT pathway inhibition.

Taken together, these observations explain why regulators and some investigators have adopted a class-wide precautionary framework. They establish biological plausibility and a shared safety concern, but they do not demonstrate that all JAKis confer the same magnitude of MACE risk. The strongest causal evidence remains specific to TOFA in a cardiovascular risk-enriched RA population; signals for other agents derive mainly from pharmacovigilance, integrated safety analyses, and less directly comparable datasets. Thus, the arguments for a class effect support class-wide vigilance, not proof of uniform class toxicity.

### 7.2 Arguments supporting a molecule-specific risk

Despite the rationale for assuming a class effect among the various JAKis, it remains difficult to accept that the cardiovascular risk profile will be similar for each drug, as evidenced by differences in the selectivity of these inhibitors. TOFA has a broader range of effects because it preferentially inhibits JAK1 and JAK3, but also inhibits JAK2 and TYK2. In contrast, baricitinib primarily acts on JAK1 and JAK2, while UPA is designed to be highly selective for JAK1 alone (McInnes et al., 2019; Traves et al., 2021; Taylor et al., 2024). This has substantial biological significance because JAK1 and JAK2 are not functionally equivalent. JAK2 is more closely associated with hematopoiesis and thrombopoietic signaling and is involved in pathways affected by IL-3, GM-CSF, and G-CSF. If inhibition of JAK1 has reached a new equilibrium between its anti-inflammatory activities and unintended vascular effects, this provides mechanistic possibilities to explain the heterogeneity observed in the cardiovascular risk of different compounds (McInnes et al., 2019; Traves et al., 2021).

Caution is warranted against overstating selectivity. *In vitro* and cellular studies suggest that FDA-approved JAKis have very similar anti-cytokine activity, particularly regarding JAK1-mediated pathways (e.g., IL-6, interferon signaling) (Virtanen et al., 2023). Additionally, while *in vitro* selectivity for a kinase does not guarantee *in vivo* specificity, therapeutic exposures exceeding certain concentration thresholds may result in inhibition beyond the selective isoform profile (Kubo et al., 2023). Therefore, kinase selectivity should be regarded as a potentially biologically relevant source of risk for a particular molecule, rather than as an established discriminative factor in clinical practice.

The complexity of dose-response relationships is further confounded by variability across drugs and dosages. If cardiovascular toxicity were solely due to a simple class effect, all drugs in this class would be expected to produce a consistent risk profile, regardless of dose. However, this theory is contradicted by existing literature. For example, in the ORAL Surveillance study comparing two dosages of TOFA (10 mg and 5 mg) for MACE, the 10 mg dose demonstrated a noninferior effect compared to 5 mg twice daily, and no discernible effect of dose on MACE was detected (Ytterberg et al., 2022). This weakens a straightforward dose-response interpretation for arterial events. However, the pattern differed for venous outcomes: adjudicated VTE and death were more frequent with TOFA 10 mg twice daily than with TNFis, and pulmonary embolism and VTE occurred more often with 10 mg than with 5 mg, prompting mid-trial dose reduction (Ytterberg et al., 2022; Di Matteo et al., 2023). In contrast, for baricitinib, evidence of dose dependence is relatively clear, with meta-analytic evidence supporting lower rates of cardiovascular disease with 2 mg versus 4 mg doses (Xie et al., 2019). For UPA, evidence for a consistent dose-response relationship in RCTs has not been demonstrated (Taylor et al., 2024). These inconsistencies do not exclude a class effect, but they suggest that pharmacologic exposure may influence risk differently across molecules and outcomes.

Interpretation is further complicated by major differences in trial populations. The ORAL Surveillance study included a clearly defined group of patients with RA, enrolling only those aged 50 years or older with at least one additional risk factor for heart disease. The mean observation period was 4.0 years, with adjudicated endpoints (Ytterberg et al., 2022). This design was deliberately intended to detect cardiovascular events. In contrast, the major development programs for baricitinib and UPA have generally included younger or minimally enriched populations, typically had shorter PBO exposure periods, used more heterogeneous background medications, and had a much lower baseline burden of cardiovascular disease (Xie et al., 2019; Genovese et al., 2018; Wang et al., 2020). Under these conditions, cross-study comparisons become inherently unstable. Lower event rates in non-TOFA programs may reflect lower underlying risk, shorter exposure, and reduced statistical power rather than true pharmacologic safety superiority.

This issue becomes especially important when analyzing the ORAL Surveillance population in more detail. Post hoc analysis showed that most of the excess cardiovascular risk occurred in patients with a prior history of atherosclerosis, older age, and a history of smoking. However, lower-risk populations do not appear to have as significant a difference in cardiovascular risk levels (Charles-Schoeman et al., 2023). This indicates that the appearance of a detectable signal is highly dependent on each patient’s vulnerability. Accordingly, the absence of a strong signal in lower-risk baricitinib or UPA cohorts cannot be interpreted as proof of equivalent safety across the class, nor can it be taken as reliable evidence that one agent is safer than another. The populations are simply too different to support a clear causal comparison.

Statistical uncertainty in non-TOFA datasets further limits interpretation. Integrated safety analyses for baricitinib and UPA are often described as reassuring, but they are based on relatively small numbers of cardiovascular events (Genovese et al., 2018; Smolen et al., 2019; Cohen et al., 2021; Burmester et al., 2025). In baricitinib datasets, MACE and VTE incidence rates were low, yet absolute event counts remained limited and formal comparisons often lacked the precision needed for strong inference (Smolen et al., 2019; Shi et al., 2024). UPA datasets show the same general pattern: numerically low rates, few events, and limited power for robust discrimination against comparators or between doses (Cohen et al., 2021; Burmester et al., 2025; Shi et al., 2024). The problem is not that the findings are negative, but that they remain statistically imprecise.

Meta-analyses have shown clear inconsistency between odds ratios for cardiovascular events associated with individual JAKis and their respective confidence intervals, which encompass both the possibility of an individual drug being beneficial and the possibility of it being harmful (Xie et al., 2019). Such estimates are not adequate to suggest equivalency but rather reflect uncertainty about the true effect size of each drug. Conversely, ORAL Surveillance has had more events, longer follow-up, and more thorough endpoint adjudication than any other non-TOFA program to date (Fleischmann et al., 2019). Even in that setting, several estimates remained imprecise. It follows that the current evidence base is still underpowered for definitive molecule-to-molecule discrimination (Misra et al., 2023; Yang et al., 2023).

Overall, the data suggest heterogeneity in cardiovascular risk among agents in the JAKi class. Differences in selectivity, dosing, exposure patterns, and study populations all provide credible reasons to suspect heterogeneity between compounds. Yet the current evidence is not strong enough to define the magnitude of those differences with confidence. Although TOFA demonstrates a significant CV safety signal, there is insufficient data to support such conclusions for baricitinib and UPA due to the lack of homogeneity in available clinical trials and imprecision. The most defensible interpretation at present is not that molecule-specific safety differences have been established, but that the available data leave genuine room for them. Until more comparable long-term datasets become available, patient-level cardiovascular risk stratification remains more evidence-based than preferential selection of one JAKi over another on the assumption of proven cardiovascular safety advantage.

### 7.3 A unifying interpretation

The connection between JAKis and MACE remains complex and not fully understood. Current evidence indicates that CV risk is heterogeneous within this drug class, likely influenced by various interacting factors rather than a single mechanism (Ytterberg et al., 2022; Taylor et al., 2022).

Initial cardiovascular risk appears to be largely determined by patient-related factors. Data from the ORAL Surveillance trial showed that the increased incidence of MACE with TOFA was especially pronounced in older patients with pre-existing CV risk factors, highlighting the importance of underlying risk enrichment (Ytterberg et al., 2022). Similar factors likely apply to the entire JAKi class, as trial groups with lower baseline risk have generally experienced fewer CV events (Taylor et al., 2022; Cohen et al., 2021).

Drug selectivity may also contribute. Differences in JAK isoform selectivity (e.g., JAK1 *versus* JAK2 *versus* broader inhibition) could play a role in the heterogeneity of safety signals; however, current data do not allow for definitive conclusions regarding differential cardiovascular risk among agents (Taylor et al., 2022; EMA, 2023). Variability in cytokine pathway modulation and its effects on lipid metabolism and inflammation may be relevant, but these mechanisms are not yet well defined.

Dose-dependent effects have been suggested, particularly for TOFA, where higher doses were associated with increased cardiovascular risk in ORAL Surveillance (Ytterberg et al., 2022). It remains uncertain whether similar dose–response relationships exist for other JAKis, due to insufficient comparative data and lower event rates (Taylor et al., 2022).

The limited duration of follow-up in clinical trials and restricted long-term exposure for some agents hinder the detection of delayed or cumulative cardiovascular risks (Taylor et al., 2022; Cohen et al., 2021). Longer observational follow-up is needed to more accurately assess potential time-dependent effects.

Overall, current evidence does not support either a purely molecule-specific interpretation or a uniform class effect. A shared mechanistic and regulatory concern exists across JAKis, but the observed clinical signal appears context-dependent, with its magnitude shaped by the individual drug, dose, indication, comparator, duration of exposure, and—most consistently—baseline patient cardiovascular risk. The most defensible current position is therefore a precautionary, risk-stratified interpretation: class-wide vigilance is warranted, while equal cardiovascular risk across all molecules should not be assumed (Ytterberg et al., 2022; EMA, 2022).

## 8 Clinical implications

### 8.1 Practical considerations

In clinical practice, initiation of a JAKis should be preceded by a structured cardiovascular risk assessment that includes age, smoking status, prior ASCVD, venous thromboembolism history, hypertension, diabetes, dyslipidemia, obesity, and overall baseline cardiovascular risk burden, because the excess signal observed in ORAL Surveillance was most evident in older RA patients with increased cardiovascular risk, particularly those with established ASCVD (Ytterberg et al., 2022; Charles-Schoeman et al., 2023). Regulatory agencies have accordingly advised greater caution in patients aged 65 years or older, current or past long-term smokers, and those with cardiovascular or malignancy risk factors; in such patients, JAKis should generally be used only when no suitable alternative exists (FDA, 2021; EMA, 2023; EMA, 2025). Lipid monitoring is also essential, as JAKis can increase total cholesterol, low-density lipoprotein cholesterol, and high-density lipoprotein cholesterol. Product information recommends reassessment after treatment initiation, typically at approximately 4–8 weeks for TOFA and around 12 weeks for UPA and baricitinib, with subsequent management according to standard lipid guidelines. Shared decision-making is therefore central and should explicitly address the patient’s baseline cardiovascular risk, prior biologic exposure, expected benefit, alternative treatment options, and the distinction between the high-risk randomized ORAL Surveillance population and more heterogeneous real-world cohorts that have often shown less pronounced excess cardiovascular risk (Ytterberg et al., 2022; Charles-Schoeman et al., 2023; FDA, 2021; EMA, 2023; EMA, 2025; Hoisnard et al., 2023; Kremer et al., 2021). In patients with high cardiovascular risk, especially those with prior ASCVD, multiple uncontrolled risk factors, or a strong thromboembolic risk profile, many clinicians may reasonably favor alternative biologic agents, particularly TNFis or other non-JAK biologics, when these are suitable, given both the regulatory cautions and the possibility that TNF inhibition itself may confer some cardiovascular benefit through suppression of systemic inflammation (FDA, 2021; EMA, 2023; EMA, 2025; Barnabe et al., 2011).

### 8.2 Future directions

Despite substantial progress in characterizing the cardiovascular safety profile of JAKis, important evidence gaps remain. Future research should prioritize dedicated, adequately powered cardiovascular outcomes trials specifically designed to compare individual JAKis with bDMARDs under harmonized conditions, with prespecified adjudication of MACE. These studies should extend beyond the high-risk enrichment strategy used in ORAL Surveillance to include broader RA populations, enabling more precise estimation of absolute and relative risk across clinically relevant cardiovascular strata. In parallel, long-term pharmacovigilance with standardized exposure definitions is required to capture delayed and rare events not detectable within clinical trial timeframes. Integration of registry-based platforms across countries could facilitate real-world head-to-head comparisons with improved statistical power and reduced regional bias. Emerging approaches in precision medicine, including biomarker profiling, genetic susceptibility markers, and inflammation-driven cardiovascular risk scores, may further refine patient selection and enable individualized JAKi therapy. Ultimately, a combined strategy incorporating randomized evidence, harmonized real-world data, and mechanistic biomarkers will be essential to resolve the remaining uncertainty regarding class-wide versus molecule-specific cardiovascular risk.

## 9 Conclusion

JAKis have significantly advanced the treatment of immune-mediated inflammatory diseases, demonstrating robust and clinically meaningful efficacy. However, concerns regarding cardiovascular safety—particularly the risk of MACE—have substantially influenced their benefit–risk evaluation.

The most compelling evidence of increased cardiovascular risk comes from the ORAL Surveillance trial, conducted in patients with pre-existing elevated cardiovascular risk. In this study, TOFA was associated with a higher rate of MACE compared with TNFis (Ytterberg et al., 2022). In contrast, data for other JAKis are less consistent. Evidence from randomized trials and real-world studies has not consistently demonstrated an increased risk; however, interpretation is limited by shorter follow-up and low event rates (Fleischmann et al., 2023; Xie et al., 2019; Alves et al., 2022; Wei et al., 2023).

Taken together, current evidence does not establish a uniform class effect of equal magnitude across all JAKis. Instead, it supports a context-dependent, risk-stratified interpretation in which a shared mechanistic and regulatory concern coexists with possible molecule- and dose-related heterogeneity. Baseline cardiovascular risk appears to be the most consistent determinant of absolute event risk, while comparative evidence for individual agents remains incomplete. Accordingly, class-wide vigilance is justified, but the TOFA signal should not be automatically extrapolated as quantitatively equivalent toxicity for every JAKi.From a clinical standpoint, these findings support a pragmatic, individualized approach to treatment. Cardiovascular risk stratification before treatment initiation, along with regular monitoring, should be standard practice. For patients with high baseline cardiovascular risk, alternative strategies may be preferred, whereas JAKis remain appropriate and effective for carefully selected lower-risk patients.

Future research should prioritize long-term studies with standardized cardiovascular endpoints, alongside mechanistic investigations to identify high-risk patient subgroups. Ultimately, integrating clinical and biological risk profiling will be essential to optimize patient selection and ensure that therapeutic benefits outweigh potential cardiovascular risks.
